# Notes on Preparations for the Sick

**Published:** 1894-03

**Authors:** 


### Notes on Preparations for the Sick.

**Analgen.**—THOMAS CHRISTY & Co., London.—This is the abbreviated name given by its patentee, Dr. Vis, to a new medicine having the following full name and affinities: ortho-aethoxy-ana-monobenzoylamido-quinoline. Its therapeutic action is similar to that of antipyrin. It is said to act promptly as an anti-neuralgic, and to be free from the depressing effects of other drugs of this class. The samples sent to us are elegant preparations in tabellæ and cachets, each of which contains seven grains; the maximum dose is 45 grains daily. Dahl & Co., Bremen, are the manufacturers.

**Syrup Yerba Santa Aromatic. Essence of Calisaya.**—PARKE, DAVIS & Co., London.—"One of the most excellent properties of **Yerba Santa** is its power of completely destroying the bitter taste of quinine, and the drug is often prescribed with reference to this property alone. It contains a principle analogous to glycyrrhizin, which renders quinine in its presence as tasteless as starch. It appears to act like glycyrrhizin, by producing a peculiar impression upon the gustatory nerves; it does not, as has been stated by some, produce with the quinine an insoluble compound. Unless the mouth is thoroughly rinsed after taking the mixture, a bitter taste will gradually develop as the nerves recover from the influence of the Yerba Santa."

The **Essence of Calisaya** is an excellent preparation of bark, each ounce representing forty grains of cinchona calisaya.

**"Plastic Pills." Cachet Pills. Perles. Flexible Gelatine Capsules.**—DUNCAN, FLOCKHART & Co., Edinburgh.—Samples of these have been forwarded to us. Our only regret is that such excellent preparations cannot always be obtained at the moment when they are most required.

**Essence of Beef.** BOVRIL LIMITED, London.—**Essence of Beef.** THE VIKING FOOD & ESSENCE COMPANY.—These beef essences are prepared from the best-quality beef, extracted without addition of water at a gentle heat. They must be nutritious, and require no digestion: they are therefore useful restoratives in cases where stimulating nourishment is needed for the purpose of counteracting nervous exhaustion and other varieties of asthenia.

**Triticumina.**—A. Meaby, Reading.—Bread made from this preparation has been brought to our notice by Mr. Henry Jones, of Broadmead, Bristol. The special features in its manufacture are, that the whole of the wheat grain is utilised, and that the bran is so finely divided that it does not cause any irritation of the alimentary canal. The wheat undergoes a malting process at specially ascertained temperatures, ensuring the conversion of a considerable proportion of the starch of the grain to dextrine. The flavour is highly palatable, and the bread retains its moisture to a marked extent. The valuable constituents of the bran are rendered assimilable by its being ground extremely fine. The bread should be easy of digestion, and must have a high nutritive value in comparison with the ordinary white breads. From the point of view of the physiologist this bread is an ideal preparation, but mankind usually requires education before it duly appreciates the ideal. It should be especially useful to the corpulent and to the diabetic patient.

---

**Specialité Lime Juice Cordial. Lemon Squash.**—Feltoe and Smith, London.—All authorities appear to be of opinion that the **Lime Juice Cordial** is one of the best of its kind. The **Lemon Squash** is an improvement on the popular beverage of that name, inasmuch as all the pulpy particles of the lemon are removed. As a non-intoxicating beverage, it should become quite the favourite.

---

**L.G.B. "Soloids" of Corrosive Sublimate. Soloids of Compressed Iodic-Hydrarg. Hazeline Snow.**—Burroughs, Wellcome & Co.—The **L. G. B. Soloids** are made according to the formulæ given by Dr. Thorne Thorne in the general memorandum with regard to disinfection issued by the Local Government Board in 1892. Two of them added to a quart of water make a one-in-a-thousand solution of corrosive sublimate. We have found this form very useful in the sick-room, and as they are easily portable, the practitioner need never be at a loss for a disinfectant solution of definite strength ready to hand.

The **Iodic-Hydrarg. Soloids** are intended for the immediate preparation of antiseptic solutions for disinfection of hands and instruments, or for vaginal and other irrigation. Each is intended for half-a-pint of water. The mercuric-potassio-mercuric-iodide is said to possess several times the bactericidal strength of hydrargyrum perchloride.

The **Hazeline Snow** contains 50 per cent. of hazeline, and is a soothing, non-greasy application for burns, insect-bites, or chapped hands. It constitutes a beautiful snow-white preparation, which leaves no mark on the whitest hand a few seconds after its application; its astringent properties render it valuable in numerous affections of the skin.

**Umbilical Envelopes.** — Reynolds & Branson, Leeds.— Hitherto the baby's share in antiseptic benefits has been almost exclusively limited to receiving into the eyes a prophylactic instillation of some disinfectant lotion. In the opinion of Dr. J. H. Whitham, of Leeds, the well-ordered infant should begin its career with more aseptic or antiseptic advantages than this, and accordingly he has devised this umbilical envelope to take the place of the time-honoured rag with which the baby's cord has usually thrived well. It can do no harm; occasionally it may do good.

